# Associations of Overweight, Obesity, and Underweight With High Serum Total Cholesterol Level Over 30 Years Among the Japanese Elderly: NIPPON DATA 80, 90, and 2010

**DOI:** 10.2188/jea.JE20170229

**Published:** 2019-04-05

**Authors:** Yosuke Shibata, Toshiyuki Ojima, Mieko Nakamura, Kazuyo Kuwabara, Naoko Miyagawa, Yoshino Saito, Yasuyuki Nakamura, Yutaka Kiyohara, Hideaki Nakagawa, Akira Fujiyoshi, Aya Kadota, Takayoshi Ohkubo, Tomonori Okamura, Hirotsugu Ueshima, Akira Okayama, Katsuyuki Miura

**Affiliations:** 1 Department of Community Health and Preventive Medicine, Hamamatsu University School of Medicine, Shizuoka, Japan; 2 Department of Preventive Medicine and Public Health, Keio University School of Medicine, Tokyo, Japan; 3 Department of Public Health, Shiga University of Medical Science, Shiga, Japan; 4 Cardiovascular Epidemiology, Kyoto Women’s University, Kyoto, Japan; 5 Health and Research Institute for Lifestyle Diseases, Fukuoka, Japan; 6 Medical Research Institute, Kanazawa Medical University, Ishikawa, Japan; 7 Center for Epidemiologic Research in Asia, Shiga University of Medical Science, Shiga, Japan; 8 Department of Hygiene and Public Health, Teikyo University School of Medicine, Tokyo, Japan; 9 Research Institute of Strategy for Prevention, Tokyo, Japan

**Keywords:** body mass index, overweight, thinness, hyperlipidemias, Japan

## Abstract

**Background:**

The trend of association between overweight and high serum total cholesterol (TC) among the elderly is unclear. In addition, there is little evidence of risk of underweight for high TC. Therefore, we examined the trend of association of overweight or underweight with high TC among Japanese elderly people using nationwide population-based data.

**Methods:**

Data of the National Survey on Circulatory Disorders and National Health and Nutrition Survey for 1980, 1990, 2000, and 2010 were used in the analysis. High TC was defined as 220 mg/dL and above. For participants aged ≥50 years, sex-specific odds ratios (ORs) of overweight or underweight compared with normal body mass index participants for high TC were calculated using a logistic regression model adjusted for age, smoking, drinking, exercise, food, and treatment of hyperlipidemia.

**Results:**

A total of 5,734, 4,673, 5,059, and 2,105 participants enrolled in these surveys in 1980, 1990, 2000, and 2010, respectively. Although overweight was positively and significantly associated with high TC in 1980, the association has gradually weakened since (ORs in 1980 and 2010 were 2.44; 95% confidence interval [CI], 1.83–3.24 and 0.92; 95% CI, 0.66–1.27 among men and 1.43; 95% CI, 1.18–1.72 and 1.08; 95% CI, 0.81–1.44 among women, respectively). While underweight was inversely and significantly associated with high TC in 1980, the association also gradually weakened among women (ORs in 1980 and 2010 were 0.28; 95% CI, 0.12–0.60 and 0.37; 95% CI, 0.10–1.28 among men and 0.39; 95% CI, 0.26–0.57 and 0.96; 95% CI, 0.58–1.57 among women, respectively).

**Conclusions:**

These findings provide evidence that high TC prevention efforts must expand the target to not only overweight but also to normal and underweight people.

## INTRODUCTION

Prevalence of obesity has increased globally. Nearly one-third of the world population is obese or overweight.^1^ Over the past three decades, the proportion of overweight has increased from 19.0% to 28.7% among Japanese men.^2^ Among Japanese women, the proportions of overweight were 20.1% and 21.3% in 1984 and 2014, respectively.^2^ However, the proportion of overweight is still higher among the elderly than young participants; the proportion of overweight in participants at 20–29 years and 60–69 years of age was 20.9% and 31.2% among men and 10.4% and 24.0% among women, respectively.^2^ The aging rate of the population in Japan is the highest in the world. Therefore, overweight in the elderly is an important public health issue in Japan.

Many studies reported high serum total cholesterol (TC) to pose a higher risk of coronary heart diseases than other measures of dyslipidemia.^3^^–^^5^ Although numerous cross-sectional studies have established that overweight and obesity are risk factors for high TC,^6^^–^^12^ the longitudinal change of the associations is controversial.^10^^,^^11^ For example, a cross-sectional study showed the odd ratios (ORs) for high TC (overweight vs normal) were 1.5 and 1.9 among men and women, respectively.^10^ The study targeted American subjects aged 50–76 years at 2000–2002 and defined high TC from self-report data. On the other hand, another survey reported the ORs for high TC (overweight or obesity vs normal) were 0.9 to 1.2.^11^ The study analyzed the National Health and Nutrition Examination Survey with stratified sampling, multistage, probability cluster design among American women aged ≥55 years at 1988–1994 and defined high TC as measured serum blood lipid levels exceeding 220 mg/dL. Thus, it is unclear whether the change of associations is due to different protocols, such as sampling, high TC definitions, and others, or due to actual change. A unified protocol is needed to conduct a longitudinal analysis.

Limited data have been published on the association between underweight and high TC.^11^^,^^13^ A cross-sectional study showed underweight inversely and significantly associated with high TC.^13^ Underweight might be a protective factor for high TC, despite a high mortality risk.^14^^,^^15^ However, the longitudinal change of the association between underweight and high TC is also unclear. The long-term trends on the impact of overweight and underweight on high TC have not been studied. Therefore, we evaluated the trend of association of overweight or underweight with high TC in a Japanese elderly population using the unified national-wide population-based data set in 1980, 1990, 2000, and 2010.

## MATERIALS AND METHODS

### Study design

We performed a panel data analysis of information from 1980, 1990, 2000, and 2010.

### Setting

The present study used data from the National Integrated Project for Prospective Observation of Non-communicable Disease And Its Trends in the Aged (NIPPON DATA), which is supported by the Ministry of Health and Welfare of Japan and consists of ongoing cohort studies conducted using the National Survey on Circulatory Disorders (NSCD) and National Health and Nutrition Survey (NHNS) in 1980, 1990, and 2010. Details of NIPPON DATA have been previously reported.^16^^–^^19^ Data of the NSCD and NHNS in 2000 were additionally obtained with permission from the Ministry of Health and Welfare of Japan. The NSCD was conducted every decade from 1960 and the NHNS was conducted annually and called the National Nutritional Survey (NNS) until 2002. The NSCD was integrated into the NHNS as of 2010. Participants were selected from 300 districts throughout Japan who were aged ≥30 years. Medical examination, blood tests, and a self-administered questionnaire about lifestyle were conducted among 10,546 participants aged ≥30 years in 1980, among 8,383 participants aged ≥30 years in 1990, among 5,565 participants aged ≥30 years in 2000, and among 2,898 participants aged ≥20 years in 2010.

### Variables

Serum TC was measured using the Lieberman-Burchard direct method in 1980 and measured enzymatically in 1990, 2000, and 2010. Measuring instruments and regents of blood tests are unified in each year. Previous study confirmed high validity between the Lieberman-Burchard direct and enzymatically method.^20^ High TC was defined as 220 mg/dL and above. Public health nurses measured height and weight and calculated body mass index (BMI) as weight (kg) divided by the square of height (m). Overweight and underweight were defined as BMI ≥25.0 kg/m^2^ and <18.5 kg/m^2^, respectively.^21^ We combined obesity (BMI ≥30.0 kg/m^2^) into the overweight category because of a lower prevalence of obesity in Japan. The prevalence of obesity was only 2.1% in 1980 and 3.4% in 2010.^2^ Public health nurses obtained information about smoking, drinking, regular exercise, and treatment histories. A dietary survey was carried out employing the weighing record method in each household.^22^ Total energy intake, saturated fatty acids (SFA), monounsaturated fatty acids (MUFA), and polyunsaturated fatty acid (PUFA) were calculated.

### Statistical analysis

For participants aged ≥50 years, sex-specific ORs and 95% confidence intervals (CIs) of overweight or underweight compared with normal BMI for high TC were calculated using a logistic regression model. Model 1 was adjusted for age. Model 2 was adjusted for age, smoking status, drinking status, regular exercise, total energy intake, SFA, MUFA, PUFA, and treatment of hyperlipidemia. Statistical significance was defined as a *P*-value of <0.05. All analyses were performed using IBM SPSS Statistics 22 for Windows (SPSS Inc, Chicago, IL, USA).

### Ethics

The institutional review board of Shiga University of Medical Science approved this study (No. 12-18, 2000, No. 22-29, 2010).

## RESULTS

A total of 5,734, 4,673, 5,059, and 2,105 participants aged ≥50 years were enrolled in the NSCD and NHNS in 1980, 1990, 2000, and 2010, respectively. The number of participants in 2010 was about half that of other periods because the sample size was designed to be small when the NSCD was integrated into NHNS in 2010. The distribution of the characteristics by periods is shown in Table 1.

**Table 1.** Characteristics of subjects aged 50 years and above in the study

	1980		1990		2000		2010	
Men								
*n*	2915		2004		2313		942	
Age, years, mean (SD)	61.7	(8.8)	63.2	(8.9)	63.7	(9.5)	67.2	(9.1)
TC, mg/dL, mean (SD)	185.0	(33.5)	196.9	(37.2)	197.9	(35.2)	201.0	(33.1)
High TC^a^, *n* (%)	314	(14.3)	488	(26.4)	359	(24.7)	253	(27.4)
BMI, kg/m^2^, mean (SD)	22.1	(2.9)	22.8	(3.0)	23.3	(3.0)	23.9	(3.0)
Underweight^b^, *n* (%)	198	(9.0)	152	(7.7)	105	(5.2)	30	(3.2)
Normal^b^, *n* (%)	1639	(74.7)	1396	(70.7)	1356	(66.6)	591	(62.7)
Overweight^b^, *n* (%)	357	(16.3)	426	(21.6)	574	(28.2)	321	(34.1)
Smoking status								
Current and Ex-smoker, *n* (%)	1819	(83.1)	1539	(77.8)	1121	(64.8)	690	(73.6)
Never smoker, *n* (%)	371	(16.9)	438	(22.2)	609	(35.2)	247	(26.4)
Drinking status								
Current drinker, *n* (%)	1454	(66.4)	1076	(54.4)	920	(53.2)	670	(71.5)
Ex-drinker, *n* (%)	199	(9.1)	197	(10.0)	201	(11.6)	37	(3.9)
Never drinker, *n* (%)	537	(24.5)	704	(35.6)	609	(35.2)	230	(24.5)
Regular exercise^c^, *n* (%)	—		495	(25.0)	638	(36.9)	398	(42.4)
Food								
Total energy intake, kcal, mean (SD)	2318.3	(525.2)	2258.4	(474.8)	2168.9	(600.6)	2131.7	(540.6)
SFA, %E, mean (SD)	5.2	(1.4)	5.5	(1.4)	6.6	(2.5)	5.9	(2.3)
MUFA, %E, mean (SD)	6.9	(1.9)	7.4	(1.8)	7.6	(2.7)	7.5	(2.6)
PUFA, %E, mean (SD)	5.1	(1.4)	5.4	(1.4)	6.1	(2.0)	5.3	(1.9)
Treatment of hyperlipidemia, *n* (%)	—		73	(3.7)	165	(7.1)	140	(14.9)

Women								
*n*	2819		2669		2746		1163	
Age, years, mean (SD)	61.9	(8.7)	63.5	(9.2)	64.7	(10.2)	66.8	(9.2)
TC, mg/dL, mean (SD)	202.1	(34.1)	218.7	(38.7)	216.1	(35.1)	216.3	(35.0)
High TC^a^, *n* (%)	814	(28.9)	1145	(47.2)	859	(44.2)	482	(42.3)
BMI, kg/m^2^, mean (SD)	23.0	(3.5)	23.3	(3.4)	23.2	(3.4)	23.1	(3.4)
Underweight^b^, *n* (%)	236	(8.4)	175	(6.7)	165	(6.6)	77	(6.6)
Normal^b^, *n* (%)	1844	(65.4)	1685	(64.3)	1652	(66.3)	770	(66.4)
Overweight^b^, *n* (%)	739	(26.2)	761	(29.0)	676	(27.1)	313	(27.0)
Smoking status								
Current and Ex-smoker, *n* (%)	361	(12.8)	283	(10.8)	184	(8.3)	108	(9.3)
Never smoker, *n* (%)	2455	(87.2)	2342	(89.2)	2043	(91.7)	1051	(90.7)
Drinking status								
Current drinker, *n* (%)	454	(16.1)	115	(4.4)	156	(7.0)	341	(29.4)
Ex-drinker, *n* (%)	52	(1.8)	26	(1.0)	28	(1.3)	9	(0.8)
Never drinker, *n* (%)	2306	(82.0)	2484	(94.6)	2043	(91.7)	810	(69.8)
Regular exercise^c^, *n* (%)	—		572	(21.8)	740	(33.2)	429	(37.0)
Food								
Total energy intake, kcal, mean (SD)	1863.1	(424.3)	1803.6	(381.4)	1762.3	(503.4)	1759.4	(424.3)
SFA, %E, mean (SD)	5.6	(1.5)	5.9	(1.5)	7.1	(2.6)	6.5	(2.4)
MUFA, %E, mean (SD)	7.4	(2.1)	7.9	(1.9)	7.9	(2.8)	8.2	(2.9)
PUFA, %E, mean (SD)	5.5	(1.6)	5.8	(1.5)	6.5	(2.1)	5.7	(2.0)
Treatment of hyperlipidemia, *n* (%)	—		164	(6.3)	351	(12.8)	252	(21.7)

BMI, body mass index; MUFA, monounsaturated fatty acids; PUFA, polyunsaturated fatty acids; SD, standard deviation; SFA, saturated fatty acids; TC, total cholesterol.

^a^High TC was defined as ≥220 mg/dL.

^b^BMI was categorized into underweight (<18.5 kg/m^2^), normal (18.5–25.0 kg/m^2^), overweight (≥25.0 kg/m^2^).

^c^Regular exercise was defined as exercise ≥2 times/week and ≥30 min/session.

The ORs of overweight for high TC are shown in Table 2. Interestingly, although a significantly positive association between overweight and high TC was observed in 1980, it was not noted in 2010.

**Table 2.** Odds ratios and 95% confidence intervals for high TC^a^

		1980	1990	2000	2010
Men					
Normal^b^		1.00 (Reference)	1.00 (Reference)	1.00 (Reference)	1.00 (Reference)

Underweight^b^	Crude	0.29 (0.13–0.59)	0.77 (0.49–1.18)	0.53 (0.26–1.09)	0.40 (0.13–1.18)
	Model 1^c^	0.31 (0.15–0.64)	0.81 (0.52–1.26)	0.59 (0.29–1.23)	0.49 (0.16–1.45)
	Model 2^d^	0.28 (0.12–0.60)	0.80 (0.51–1.25)	0.63 (0.3–1.3)	0.37 (0.10–1.28)

Overweight^b^	Crude	2.50 (1.89–3.29)	2.10 (1.65–2.66)	1.51 (1.17–1.95)	0.93 (0.68–1.26)
	Model 1^c^	2.44 (1.85–3.21)	2.07 (1.62–2.62)	1.46 (1.13–1.88)	0.90 (0.65–1.22)
	Model 2^d^	2.44 (1.83–3.24)	2.01 (1.57–2.56)	1.43 (1.10–1.86)	0.92 (0.66–1.27)

Women					
Normal^b^		1.00 (Reference)	1.00 (Reference)	1.00 (Reference)	1.00 (Reference)

Underweight^b^	Crude	0.39 (0.26–0.57)	0.64 (0.45–0.90)	0.64 (0.42–0.97)	1.01 (0.62–1.62)
	Model 1^c^	0.39 (0.26–0.57)	0.65 (0.45–0.91)	0.65 (0.43–0.99)	1.03 (0.63–1.66)
	Model 2^d^	0.39 (0.26–0.57)	0.68 (0.47–0.96)	0.63 (0.41–0.96)	0.96 (0.58–1.57)

Overweight^b^	Crude	1.44 (1.20–1.72)	1.30 (1.08–1.55)	1.13 (0.93–1.38)	0.89 (0.67–1.16)
	Model 1^c^	1.43 (1.19–1.72)	1.29 (1.08–1.54)	1.15 (0.94–1.40)	0.91 (0.68–1.19)
	Model 2^d^	1.43 (1.18–1.72)	1.33 (1.10–1.59)	1.14 (0.93–1.40)	1.08 (0.81–1.44)

TC, total cholesterol.

^a^High TC was defined as ≥220 mg/dL.

^b^BMI was categorized into underweight (<18.5 kg/m^2^), normal (18.5–25.0 kg/m^2^), overweight (≥25.0 kg/m^2^).

^c^Model 1 adjusted for age.

^d^Model 2 adjusted for age, smoking status, drinking status, regular exercise, total energy intake, saturated fatty acids, monounsaturated fatty acids, polyunsaturated fatty acids, and treatment of hyperlipidemia.

In order to confirm the changes, analyses by treatment of hyperlipidemia were performed because of the common use of drug therapy for hyperlipidemia since the 1990s. Both non-treatment and treatment participants showed a decreased trend; ORs among non-treatment participants in 1980 to 2010 were 2.50 to 1.00 among men and 1.44 to 1.02 among women, and those among treatment participants decreased from 1.84 to 0.81 among men and from 1.51 to 0.91 among women (eTable 1).

The ORs of underweight for high TC are shown in Table 2. Although underweight was inversely and significantly associated with high TC in 1980, the relationship had weakened among women but not among men in 2010. In treatment-specific analysis, ORs among non-treatment and treatment women in 1980 to 2010 were 0.39 to 0.88 and 0.86 to 1.51, respectively. Among men, ORs in non-treatment participants remained, ranging from 0.29 to 0.30 from 1980 to 2010. In treatment participants, the sample size was too small for analysis. (eTable 1).

## DISCUSSION

In this large population-based study, we found that the association between overweight and high TC was gradually weakened over time, although a positive and significant association was observed in 1980. Whereas underweight was inversely and significantly associated with high TC in 1980, it was also gradually weakened among women but not men.

Numerous studies showed that overweight is positively associated with high TC.^9^^–^^11^^,^^14^^,^^19^^,^^23^ The analysis of the third National Health and Nutrition Examination Survey 1988–1994 with American participants aged ≥60 years, the ORs of BMI ≥30 kg/m^2^ versus BMI <25 kg/m^2^ were 1.5 and 1.8 among men and women, respectively.^23^ In another study, the proportion of high-TC participants was increased following weight gain in a Dutch population in 1974–1980.^14^ Our results were consistent with these previous studies and revealed that these associations weakened in a later period. The reason for the weakened associations is still unknown, but three reasons may partly explain the trend. First, high-TC participants increased among the normal BMI population (eTable 2). The proportion of high TC among normal BMI participants increased by about twofold over 3 decades (12.8% to 29.2% and 28.1% to 46.9% among men and women, respectively), whereas it only slightly increased among obese participants. In Japan, a high-fat westernized diet became increasingly popular over the study period.^24^ Fat intake increased regardless of BMI categories (Table 1). The northern Sweden MONICA study revealed that dietary factors mainly contribute to the TC level.^25^ Second, the proportion of participants who have protective factors for TC elevation has increased more among the obese compared with normal BMI participants (eTable 2). Although the differences in total energy intake between normal BMI and overweight participants were 147.6 kcal among men and 9.1 kcal among women in 1980, they became 56.8 kcal among men and −97.6 kcal among women in 2010. Furthermore, the proportion of current drinkers decreased among the overweight compared with normal BMI participants in men. In Japan, overweight has drawn much attention in the past few decades; for example, a new health examination focusing on metabolic syndrome started in 2008. The second reason reflects the spread of knowledge of the harm of being overweight. Finally, the proportion of overweight participants with well-controlled TC values in the treatment population greatly increased in spite of the fact that the proportion of treatment participants had increased by about 4 times over 3 decades (eTable 3). The proportion of high-TC participants among the overweight population decreased from approximately 60% to 20% over 2 decades. One reason is that the total energy intake among overweight persons was relatively decreased. Total energy intake declined to low or equal in 2010, whereas it was high in 1990. The final reason, perhaps, may be the development of drug therapy. These explanatory mechanisms mean not only that the proportion of high-TC participants had increased among those with normal BMI but also that the proportion of such participants had relatively decreased among overweight participants.

Limited studies have reported that underweight is inversely associated with high TC. The proportion of high TC was one-fourth to one-half among underweight compared with normal BMI participants in the United States during 1988–1990.^11^ A cross-sectional study in 1998 in Korea found that the OR of BMI <18.5 kg/m^2^ versus BMI 18.5–22.0 kg/m^2^ for high TC was 0.58 (95% CI, 0.40–0.85) among women.^13^ The present study was consistent with these previous studies and revealed that the association had weakened among women. A possible explanation for the findings is that high-TC participants had increased among underweight participants, and the proportion of high TC among underweight participants at 1980 to 2010 was 13.1% to 43.7% (eTable 2). It is well established that the proportion of underweight Japanese women is the highest among developed countries, at 11.6%.^26^ A cross-sectional study report that participants low in weight were not satisfied with their body among Japanese women compared with findings in other Asian participants.^27^ However, interestingly, underweight Japanese women increased their fat intake. Thus, the proportion of high-TC participants among underweight Japanese women has increased due to a fatty diet.

A main advantage of this study is the use of unified national population-based data in 1980, 1990, 2000, and 2010. Moreover, potential confounding factors, such as smoking,^28^ drinking,^29^ exercise^30^^,^^31^ and food,^32^^,^^33^ were adjusted. To our knowledge, this is the first study to reveal the change of associations between overweight or underweight and high TC over 30 years using a consistent protocol.

However, our study has limitations. First, the results were only from Japanese elderly. Several studies reported that body composition in Asia is largely different from a Western population.^34^^–^^36^ The proportion of body fat at any given BMI among Asian participants tends to be a higher than among caucasians.^36^^,^^37^ WHO mentioned that the population-specific cut-off point for BMI is necessary.^38^ More studies are needed to clarify the association among young people or a Western population. Second, the response rate has been decreasing over time in national surveys in Japan. Selection bias might be possible. Third, the mean age has increased. Although we adjusted for age in each survey, the effect of age on health status might be included. Fourth, high TC was defined as 220 mg/dL and above. According to WHO categories, high TC was defined as 200 mg/dL and above. But the risk of coronary artery diseases is lower among Japanese than Western participants.^39^ The Guidelines for the Diagnosis and Prevention of Atherosclerosis Cardiovascular Diseases by the Japan Atherosclerosis Society in 1997 have indicated that TC value for treatment was serum blood lipid levels ≥220 mg/dL.^40^ Therefore, we defined high TC as measured serum blood lipid levels ≥220 mg/dL. Fifth, TC measurement methods differed across 1980, 1990, 2000, and 2010 because the method of enzymatically measurement was not popular in 1980. Finally, other serum lipids, such as low-density lipoprotein cholesterol, high-density lipoprotein cholesterol, and triglyceride were not used for the outcomes. The TC value was only an item when the serum lipid profile was assessed in 1980. For the longitudinal association analysis, the TC value was used as an outcome in the present study.

### Conclusions

The association between overweight and high TC was gradually weakened, although it was positively and significantly associated both in men and women. The association between underweight and high TC was also gradually weakened among women, whereas it was inversely and significantly associated both in men and women. These findings provide evidence that high TC prevention efforts, nowadays, must expand their target to not only overweight but also normal and underweight participants.

## APPENDIX A. SUPPLEMENTARY DATA

eTable 1. Treatment-specific odds ratios and 95% confidence intervals for high TC^a^

eTable 2. Characteristics of not-treated participants by BMI categories

eTable 3. Characteristics of treated participants by BMI categories

## APPENDIX B.

### The NIPPON DATA80/90/2010 Research Group

Chairpersons: NIPPON DATA80/90: Hirotsugu Ueshima (Center for Epidemiologic Research in Asia, Shiga University of Medical Science, Shiga, Japan). NIPPON DATA2010: Katsuyuki Miura (Department of Public Health, Shiga University of Medical Science, Shiga, Japan). Co-chairpersons: NIPPON DATA80/90: Akira Okayama (Research Institute of Strategy for Prevention, Tokyo) for the NIPPON DATA80, and Tomonori Okamura (Department of Preventive Medicine and Public Health, Keio University, Tokyo) for the NIPPON DATA90. NIPPON DATA2010: Akira Okayama (Research Institute of Strategy for Prevention, Tokyo), and Tomonori Okamura (Department of Preventive Medicine and Public Health, Keio University, Tokyo). Participating researchers: Hirotsugu Ueshima (Department of Public Health, Shiga University of Medical Science, Otsu, Shiga), Akira Okayama (Research Institute of Strategy for Prevention, Tokyo), Tomonori Okamura (Department of Preventive Medicine and Public Health, School of Medicine, Keio University, Tokyo), Shigeyuki Saitoh (Sapporo Medical University School of Health Science, Sapporo, Hokkaido), Kiyomi Sakata (Department of Hygiene and Preventive Medicine, Iwate Medical University, Morioka, Iwate), Atsushi Hozawa (Department of Preventive Medicine and Epidemiology, Tohoku Medical Megabank Organization, Tohoku University, Sendai, Miyagi), Takehito Hayakawa (Department of Hygiene and Preventive Medicine, Fukushima Medical University, School of Medicine, Fukushima), Yosikazu Nakamura (Department of Public Health, Jichi Medical University, Shimotsuke, Tochigi), Nobuo Nishi (Center for International Collaboration and Partnership National Institute of Health and Nutrition National Institutes of Biomedical Innovation, Health and Nutrition, Tokyo), Nagako Okuda (Department of Health and Nutrition, University of Human Arts and Sciences, Saitama), Tomonori Okamura (Department of Preventive Medicine and Public Health, Keio University, Tokyo), Yoshitaka Murakami (Department of Medical Statistics, Toho University School of Medicine, Tokyo), Takayoshi Ohkubo (Department of Hygiene and Public Health, Teikyo University School of Medicine, Tokyo), Fumiyoshi Kasagi (Institute of Radiation Epidemiology, Radiation Effects Association, Tokyo), Toru Izumi (Faculty of Medicine, Kitasato University, Sagamihara, Kanagawa), Yasuhiro Matsumura (Faculty of Health and Nutrition, Bunkyo University, Chigasaki, Kanagawa), Toshiyuki Ojima (Department of Community Health and Preventive Medicine, Hamamatsu University School of Medicine, Hamamatsu, Shizuoka), Koji Tamakoshi (Department of Public Health and Health Information Dynamics, Nagoya University Graduate School of Medicine, Nagoya, Aichi), Hideaki Nakagawa (Medical Research Institute, Kanazawa Medical University, Kanazawa, Ishikawa), Yoshikuni Kita (Faculty of Nursing Science, Department of Nursing Science, Tsuruga Nursing University, Tsuruga), Katsuyuki Miura, Aya Kadota (Department of Public Health, Shiga University of Medical Science, Otsu, Shiga), Naomi Miyamatsu (Department of Clinical Nursing Science Lecture, Shiga University of Medical Science, Otsu, Shiga), Yasuyuki Nakamura (Cardiovascular Epidemiology, Kyoto Women’s University, Kyoto), Katsushi Yoshita (Osaka City University Graduate School of Human Life Science, Osaka), Yoshihiro Miyamoto (Department of Preventive Cardiology, National Cerebral and Cardiovascular Center, Suita, Osaka), Kazunori Kodama (Radiation Effects Research Foundation, Hiroshima) and Yutaka Kiyohara (Department of Environmental Medicine, Kyushu University, Fukuoka).

## References

[1] Ng M, Fleming T, Robinson M, Global, regional, and national prevalence of overweight and obesity in children and adults during 1980–2013: a systematic analysis for the Global Burden of Disease Study 2013. Lancet. 2014;384:766–781. 10.1016/S0140-6736(14)60460-8 24880830 PMC4624264

[2] Office of Nutrition, Cancer Measures and Health Promotion Division, Health Service Bureau, Ministry of Health, Labour and Welfare, Japan. The National Health and Nutrition Survey in Japan, 2014. http://www.mhlw.go.jp/bunya/kenkou/eiyou/dl/h26-houkoku.pdf (accessed November 15, 2017).

[3] Okamura T, Tanaka H, Miyamatsu N, Hayakawa T, Kadowaki T, Kita Y; NIPPON DATA80 Research Group The relationship between serum total cholesterol and all-cause or cause-specific mortality in a 17.3-year study of a Japanese cohort. Atherosclerosis. 2007;190:216–223. 10.1016/j.atherosclerosis.2006.01.024 16529754

[4] Peters SAE, Singhateh Y, Mackay D, Huxley RR, Woodward M Total cholesterol as a risk factor for coronary heart disease and stroke in women compared with men: a systematic review and meta-analysis. Atherosclerosis. 2016;248:123–131. 10.1016/j.atherosclerosis.2016.03.016 27016614

[5] Nagasawa SY, Okamura T, Iso H, Tamakoshi A, Yamada M, Watanabe M; Evidence for Cardiovascular Prevention from Observational Cohorts in Japan (EPOCH-JAPAN) Research Group Relation between serum total cholesterol level and cardiovascular disease stratified by sex and age group: a pooled analysis of 65 594 individuals from 10 cohort studies in Japan. J Am Heart Assoc. 2012;1:e001974. 10.1161/JAHA.112.001974 23316288 PMC3541631

[6] Kawada T Body mass index is a good predictor of hypertension and hyperlipidemia in a rural Japanese population. Int J Obes Relat Metab Disord. 2002;26:725–729. 10.1038/sj.ijo.0801984 12032759

[7] Kearns K, Dee A, Fitzgerald AP, Doherty E, Perry IJ Chronic disease burden associated with overweight and obesity in Ireland: the effects of a small BMI reduction at population level. BMC Public Health. 2014;14:143. 10.1186/1471-2458-14-143 24512151 PMC3929131

[8] Kress AM, Hartzel MC, Peterson MR Burden of disease associated with overweight and obesity among U.S. military retirees and their dependents, aged 38–64, 2003. Prev Med. 2005;41:63–69. 10.1016/j.ypmed.2004.10.012 15916994

[9] Janssen I, Katzmarzyk PT, Ross R Body mass index, waist circumference, and health risk: evidence in support of current National Institutes of Health guidelines. Arch Intern Med. 2002;162:2074–2079. 10.1001/archinte.162.18.2074 12374515

[10] Patterson RE, Frank LL, Kristal AR, White E A comprehensive examination of health conditions associated with obesity in older adults. Am J Prev Med. 2004;27:385–390. 10.1016/j.amepre.2004.08.001 15556738

[11] Must A, Spadano J, Coakley EH, Field AE, Colditz G, Dietz WH The disease burden associated with overweight and obesity. JAMA. 1999;282:1523–1529. 10.1001/jama.282.16.1523 10546691

[12] Manson JE, Colditz GA, Stampfer MJ, A prospective study of obesity and risk of coronary heart disease in women. N Engl J Med. 1990;322:882–889. 10.1056/NEJM199003293221303 2314422

[13] Kim Y, Suh YK, Choi H BMI and metabolic disorders in South Korean adults: 1998 Korea National Health and Nutrition Survey. Obes Res. 2004;12:445–453. 10.1038/oby.2004.50 15044661

[14] Seidell JC, Verschuren WM, van Leer EM, Kromhout D Overweight, underweight, and mortality: a prospective study of 48,287 men and women. Arch Intern Med. 1996;156:958–963. 10.1001/archinte.1996.00440090054006 8624176

[15] Adams KF, Schatzkin A, Harris TB, Overweight, obesity, and mortality in a large prospective cohort of persons 50 to 71 years old. N Engl J Med. 2006;355:763–778. 10.1056/NEJMoa055643 16926275

[16] Hisamatsu T, Miura K, Ohkubo T, Yamamoto T, Fujiyoshi A, Miyagawa N; NIPPON DATA80 Research Group Interaction between dietary marine-derived n-3 fatty acids intake and J-point elevation on the risk of cardiac death: a 24-year follow-up of Japanese men. Heart. 2013;99:1024–1029. 10.1136/heartjnl-2012-303496 23666393

[17] Kadota A, Hozawa A, Okamura T, Kadowak T, Nakmaura K, Murakami Y; NIPPON DATA Research Group Relationship between metabolic risk factor clustering and cardiovascular mortality stratified by high blood glucose and obesity NIPPON DATA90, 1990–2000. Diabetes Care. 2007;30:1533–1538. 10.2337/dc06-2074 17363755

[18] Kadowaki S, Okamura T, Hozawa A, Kadowaki T, Kadota A, Murakami Y; NIPPON DATA Research Group Relationship of elevated casual blood glucose level with coronary heart disease, cardiovascular disease and all-cause mortality in a representative sample of the Japanese population. NIPPON DATA80. Diabetologia. 2008;51:575–582. 10.1007/s00125-007-0915-6 18197396

[19] Hozawa A, Okamura T, Oki I, Murakami Y, Kadowaki T, Nakamura K; NIPPON DATA80 Study Group Relationship between BMI and all-cause mortality in Japan: NIPPON DATA80. Obesity. 2008;16:1714–1717. 10.1038/oby.2008.237 18421264

[20] Naka H Change of method for determination of serum total cholesterol and verification of it’s accuracy over the past thirty years. Official J Jpn Soc Hum Dry Dock. 2001;16:211–215 (in Japanese) 10.11320/ningendock1986.16.211

[21] Examination Committee of Criteria for ‘Obesity Disease’ in Japan; Japan Society for the Study of Obesity New criteria for ‘obesity disease’ in Japan. Circ J. 2002;66:987–992. 10.1253/circj.66.987 12419927

[22] Okuda N, Miura K, Yoshita K, Matsumura Y, Okayama A, Nakamura Y; NIPPON DATA80/90 Research Group Integration of data from NIPPON DATA80/90 and National Nutrition Survey in Japan: for cohort studies of representative Japanese on nutrition. J Epidemiol. 2010;20:S506–S514. 10.2188/jea.JE20090218 20351471 PMC3920387

[23] Brown CD, Higgins M, Donato KA, Body mass index and the prevalence of hypertension and dyslipidemia. Obes Res. 2000;8:605–619. 10.1038/oby.2000.79 11225709

[24] Egusa G, Murakami F, Ito C, Westernized food habits and concentrations of serum lipids in the Japanese. Atherosclerosis. 1993;100:249–255. 10.1016/0021-9150(93)90211-C 8357357

[25] Eliasson M, Janlert U, Jansson JH, Stegmayr B Time trends in population cholesterol levels 1986–2004: influence of lipid-lowering drugs, obesity, smoking and educational level. The northern Sweden MONICA study. J Intern Med. 2006;260:551–559. 10.1111/j.1365-2796.2006.01730.x 17116006

[26] Office of Nutrition, Cancer Measures and Health Promotion Division, Health Service Bureau, Ministry of Health, Labour and Welfare, Japan. Summary results of the National Health and Nutrition Survey in Japan, 2016. http://www.mhlw.go.jp/file/04-Houdouhappyou-10904750-Kenkoukyoku-Gantaisakukenkouzoushinka/kekkagaiyou_7.pdf (accessed November 15, 2017).

[27] Yates A, Edman J, Aruguete M Ethnic differences in BMI and body/self-dissatisfaction among Whites, Asian subgroups, Pacific Islanders, and African-Americans. J Adolesc Health. 2004;34:300–307. 10.1016/S1054-139X(03)00305-7 15040999

[28] Tucker LA Use of smokeless tobacco, cigarette smoking, and hypercholesterolemia. Am J Public Health. 1989;79:1048–1050. 10.2105/AJPH.79.8.1048 2751026 PMC1349909

[29] Brien SE, Ronksley PE, Turner BJ, Mukamal KJ, Ghali WA Effect of alcohol consumption on biological markers associated with risk of coronary heart disease: systematic review and meta-analysis of interventional studies. BMJ. 2011;342:d636. 10.1136/bmj.d636 21343206 PMC3043110

[30] Weiss EP, Albert SG, Reeds DN, Effects of matched weight loss from calorie restriction, exercise, or both on cardiovascular disease risk factors: a randomized intervention trial. Am J Clin Nutr. 2016;104:576–586. 10.3945/ajcn.116.131391 27465384 PMC4997297

[31] Bischoff SC, Damms-Machado A, Betz C, Multicenter evaluation of an interdisciplinary 52-week weight loss program for obesity with regard to body weight, comorbidities and quality of life—a prospective study. Int J Obes. 2012;36:614–624. 10.1038/ijo.2011.107 21673653 PMC3322430

[32] Petersen M, Taylor MA, Saris WH, Randomized, multi-center trial of two hypo-energetic diets in obese subjects: high-versus low-fat content. Int J Obes. 2006;30:552–560. 10.1038/sj.ijo.0803186 16331300

[33] Chowdhury R, Warnakula S, Kunutsor S, Association of dietary, circulating, and supplement fatty acids with coronary risk: a systematic review and meta-analysis. Ann Intern Med. 2014;160:398–406. 10.7326/M13-1788 24723079

[34] Seidell JC, Kahn HS, Williamson DF, Lissner L, Valdez R Report from a Centers for Disease Control and Prevention Workshop on use of adult anthropometry for public health and primary health care. Am J Clin Nutr. 2001;73:123–126. 10.1093/ajcn/73.1.123 11124761

[35] Deurenberg P, Yap M, van Staveren WA Body mass index and percent body fat: a meta analysis among different ethnic groups. Int J Obes Relat Metab Disord. 1998;22:1164–1171. 10.1038/sj.ijo.0800741 9877251

[36] Chang CJ, Wu CH, Chang CS, Low body mass index but high percent body fat in Taiwanese subjects: implications of obesity cutoffs. Int J Obes Relat Metab Disord. 2003;27:253–259. 10.1038/sj.ijo.802197 12587007

[37] Wang J, Thornton JC, Russell M, Burastero S, Heymsfield S, Pierson RN Jr Asians have lower body mass index (BMI) but higher percent body fat than do whites: comparisons of anthropometric measurements. Am J Clin Nutr. 1994;60:23–28. 10.1093/ajcn/60.1.23 8017333

[38] WHO Expert Consultation Appropriate body-mass index for Asian populations and its implications for policy and intervention strategies. Lancet. 2004;363:157–163. 10.1016/S0140-6736(03)15268-3 14726171

[39] Saito I, Folsom AR, Aono H, Ozawa H, Ikebe T, Yamashita T Comparison of fatal coronary heart disease occurrence based on population surveys in Japan and the USA. Int J Epidemiol. 2000;29:837–844. 10.1093/ije/29.5.837 11034966

[40] Japan Atherosclerosis Society. *Guideline for the Diagnosis and Prevention of Atherosclerosis Cardiovascular Diseases*. 1997.

