# Supplementary material for: Associations of Overweight, Obesity, and Underweight With High Serum Total Cholesterol Level Over 30 Years Among the Japanese Elderly: NIPPON DATA 80, 90, and 2010
Source: J Epidemiol. 2019 Apr 5;29(4):133–8. doi: 10.2188/jea.JE20170229 (PMC6414806; doi:10.2188/jea.JE20170229)
Supplement: Supplementary file 1 [file je-29-133-s001.pdf]

eTable 1. Treatment-specific odds ratios and 95% confidence intervals for high TC[a]

				1980	1990	2000	2010
Non-treatment							
	Men	Normal[b]		1.00 (Reference)	1.00 (Reference)	1.00 (Reference)	1.00 (Reference)
		Underweight[b]	Crude	0.29 (0.14–0.59)	0.74 (0.47–1.16)	0.50 (0.23–1.05)	0.30 (0.08–0.99)
			Model 1[c]	0.31 (0.15–0.64)	0.80 (0.50–1.26)	0.56 (0.26–1.20)	0.37 (0.10–1.27)
			Model 2[d]	0.28 (0.12–0.60)	0.77 (0.48–1.21)	0.56 (0.26–1.21)	0.24 (0.05–1.06)
		Overweight[b]	Crude	2.50 (1.89–3.29)	2.04 (1.58–2.61)	1.55 (1.18–2.02)	1.00 (0.71–1.38)
			Model 1[c]	2.44 (1.85–3.21)	1.99 (1.55–2.55)	1.48 (1.13–1.94)	0.96 (0.68–1.33)
			Model 2[d]	2.44 (1.83–3.24)	2.01 (1.56–2.58)	1.49 (1.13–1.96)	0.92 (0.65–1.30)
	Women	Normal[b]		1.00 (Reference)	1.00 (Reference)	1.00 (Reference)	1.00 (Reference)
		Underweight[b]	Crude	0.39 (0.26–0.57)	0.65 (0.45–0.92)	0.63 (0.40–0.98)	0.88 (0.53–1.44)
			Model 1[c]	0.39 (0.26–0.57)	0.67 (0.46–0.95)	0.66 (0.42–1.03)	0.90 (0.54–1.48)
			Model 2[d]	0.39 (0.26–0.57)	0.68 (0.47–0.97)	0.63 (0.39–0.98)	0.93 (0.55–1.55)
		Overweight[b]	Crude	1.44 (1.20–1.72)	1.24 (1.03–1.49)	1.11 (0.89–1.38)	1.02 (0.74–1.40)
			Model 1[c]	1.43 (1.19–1.72)	1.24 (1.03–1.49)	1.14 (0.91–1.42)	1.05 (0.76–1.43)
			Model 2[d]	1.43 (1.18–1.72)	1.32 (1.09–1.59)	1.16 (0.92–1.44)	1.11 (0.79–1.54)
Treatment							
	Men	Normal[b]		–	N/A	N/A	1.00 (Reference)
		Underweight[b]	Crude	–	N/A	N/A	4.38 (0.25–74.7)
			Model 1[c]	–	N/A	N/A	8.02 (0.40–159.)
			Model 2[d]	–	N/A	N/A	9.45 (0.44–202.)
		Overweight[b]	Crude	–	1.84 (0.65–5.15)	0.93 (0.40–2.15)	0.81 (0.32–2.00)
			Model 1[c]	–	1.96 (0.68–5.63)	0.99 (0.42–2.31)	0.78 (0.31–1.94)
			Model 2[d]	–	2.45 (0.76–7.82)	1.09 (0.44–2.63)	0.84 (0.31–2.23)
	Women	Normal[b]		–	1.00 (Reference)	1.00 (Reference)	1.00 (Reference)
		Underweight[b]	Crude	–	0.86 (0.13–5.43)	0.70 (0.21–2.28)	1.51 (0.26–8.61)
			Model 1[c]	–	0.83 (0.13–5.21)	0.69 (0.20–2.29)	1.58 (0.27–9.07)
			Model 2[d]	–	0.65 (0.08–4.97)	0.93 (0.25–3.40)	1.60 (0.26–9.63)
		Overweight[b]	Crude	–	1.51 (0.74–3.08)	1.10 (0.66–1.83)	0.91 (0.49–1.67)
			Model 1[c]	–	1.49 (0.72–3.05)	1.15 (0.68–1.92)	0.90 (0.49–1.66)
			Model 2[d]	–	1.65 (0.77–3.51)	1.19 (0.70–2.01)	1.03 (0.54–1.94)

N/A, not available because sample size was too small; TC, total cholesterol.

[a]High TC was defined as ≥220 mg/dL.

[b]BMI was categorized into underweight (<18.5 kg/m^2), normal (18.5–25.0 kg/m^2), overweight (≥25.0 kg/m^2).

[c]Model 1 adjusted for age.

[d]Model 2 adjusted for age, smoking status, drinking status, regular exercise, total energy intake, saturated fatty acids, monounsaturated fatty acids, and polyunsaturated fatty acids.

eTable 2. Characteristics of not-treated participants by BMI categories

Variables	1980			1990			2000			2010		
	Underweight[a]	Normal[a]	Overweight[a]	Underweight[a]	Normal[a]	Overweight[a]	Underweight[a]	Normal[a]	Overweight[a]	Underweight[a]	Normal[a]	Overweight[a]
Men												
n	198	1639	357	150	1342	395	104	1264	516	28	518	255
Age, years, mean (SD)	66.8 (9.4)	61.6 (8.7)	59.5 (8.1)	67.8 (8.9)	63.1 (8.9)	61.6 (8.4)	68.6 (11.5)	63.8 (9.4)	62.2 (8.3)	73.0 (9.5)	67.0 (8.9)	66.1 (9.3)
TC, mg/dL, mean (SD)	172.5 (27.3)	183.7 (33.0)	198.4 (34.9)	179.5 (36.1)	194.2 (35.0)	207.3 (38.2)	185.8 (34.7)	195.0 (33.9)	203.7 (34.7)	185.6 (36.3)	204.2 (33.1)	202.4 (30.9)
High TC[b], n (%)	8 (4.1)	210 (12.8)	96 (26.9)	25 (16.7)	282 (21.0)	139 (35.2)	8 (12.1)	196 (21.8)	116 (30.1)	≤5	151 (29.2)	75 (29.4)
BMI, kg/m^2, mean (SD)	17.5 (0.9)	21.7 (1.7)	26.8 (1.5)	17.4 (0.9)	22.0 (1.7)	26.9 (1.8)	17.5 (0.9)	22.2 (1.7)	26.9 (1.9)	17.5 (0.7)	22.4 (1.7)	26.9 (1.6)
Smoking status												
Current and Ex-smoker, n (%)	179 (91.3)	1375 (84.0)	264 (74.2)	121 (80.7)	1038 (77.3)	313 (79.2)	47 (54.7)	467 (43.9)	153 (33.9)	16 (57.1)	374 (72.8)	187 (73.6)
Never smoker, n (%)	17 (8.7)	262 (16.0)	92 (25.8)	29 (19.3)	304 (22.7)	82 (20.8)	39 (45.3)	597 (56.1)	298 (66.1)	12 (42.9)	140 (27.2)	67 (26.4)
Drinking status												
Current drinker, n (%)	110 (55.8)	1092 (66.7)	251 (70.7)	60 (40.0)	747 (55.7)	230 (58.2)	34 (40.0)	574 (53.9)	255 (56.7)	N/A	378 (73.5)	179 (70.5)
Ex-drinker, n (%)	26 (13.2)	146 (8.9)	27 (7.6)	24 (16.0)	120 (8.9)	37 (9.4)	13 (15.3)	119 (11.2)	49 (10.9)	N/A	20 (3.9)	11 (4.3)
Never drinker, n (%)	61 (31.0)	399 (24.4)	77 (21.7)	66 (44.0)	475 (35.4)	128 (32.4)	38 (44.7)	371 (34.9)	146 (32.4)	N/A	116 (22.6)	64 (25.2)
Regular exercise[c], n (%)	-	-	-	35.0 (23.3)	343.0 (25.6)	94.0 (23.8)	23 (27.1)	396 (37.3)	152 (33.9)	8 (28.6)	231 (44.8)	93 (36.6)
Food												
Total energy intake, kcal, mean (SD)	2147.5 (443.9)	2308.9 (512.4)	2456.5 (587.9)	2132.4 (459.1)	2263.3 (474.1)	2327.2 (474.4)	1961.8 (508.1)	2187.2 (600.0)	2260.0 (590.7)	1765.1 (429.4)	2140.9 (528.4)	2197.7 (588.6)
SFA, %E, mean (SD)	5.1 (1.3)	5.1 (1.4)	5.3 (1.3)	5.5 (1.5)	5.5 (1.3)	5.5 (1.3)	6.3 (2.8)	6.6 (2.5)	6.6 (2.4)	5.7 (2.3)	5.8 (2.3)	5.9 (2.3)
MUFA, %E, mean (SD)	6.6 (1.8)	6.9 (1.9)	7.2 (2.0)	7.4 (2.0)	7.4 (1.8)	7.5 (1.7)	7.1 (2.7)	7.6 (2.8)	7.7 (2.8)	6.8 (2.7)	7.5 (2.6)	7.7 (2.7)
PUFA, %E, mean (SD)	4.8 (1.3)	5.1 (1.4)	5.3 (1.4)	5.4 (1.5)	5.4 (1.3)	5.6 (1.4)	5.7 (2.1)	6.1 (1.9)	6.2 (2.0)	5.1 (2.0)	5.2 (1.8)	5.4 (2.1)
Women												
n	236	1844	739	169	1579	687	152	1453	550	71	623	215
Age, years, mean (SD)	63.7 (9.3)	62.0 (8.8)	61.0 (8.3)	67.5 (9.6)	63.0 (9.4)	62.8 (8.6)	67.6 (12.1)	63.2 (9.9)	64.3 (9.2)	66.3 (10.3)	65.6 (9.1)	66.4 (9.5)
TC, mg/dL, mean (SD)	188.5 (30.5)	201.2 (33.7)	208.6 (34.5)	209.2 (35.5)	215.4 (37.9)	222.0 (38.0)	208.5 (30.5)	215.2 (35.1)	217.5 (36.7)	216.9 (32.9)	220.3 (34.2)	221.3 (40.1)
High TC[b], n (%)	31 (13.1)	517 (28.1)	266 (36.0)	51 (30.2)	655 (41.5)	320 (46.6)	31 (32.3)	483 (43.0)	208 (45.6)	31 (43.7)	292 (46.9)	99 (46.0)
BMI, kg/m^2, mean (SD)	17.2 (1.1)	22.0 (1.7)	27.5 (2.1)	17.2 (1.0)	22.0 (1.7)	27.3 (2.1)	17.3 (1.0)	22.0 (1.8)	27.5 (2.3)	17.4 (0.9)	21.9 (1.7)	27.4 (2.2)
Smoking status												
Current and Ex-smoker, n (%)	51 (21.7)	215 (11.7)	95 (12.9)	25 (14.8)	166 (10.5)	61 (8.9)	11 (9.2)	94 (7.3)	31 (6.2)	11 (15.5)	58 (9.4)	20 (9.3)
Never smoker, n (%)	184 (78.3)	1627 (88.3)	644 (87.1)	144 (85.2)	1413 (89.5)	626 (91.1)	109 (90.8)	1190 (92.7)	473 (93.8)	60 (84.5)	561 (90.6)	195 (90.7)
Drinking status												
Current drinker, n (%)	N/A	310 (16.9)	107 (14.5)	N/A	80 (5.1)	25 (3.6)	N/A	94 (7.3)	33 (6.6)	18 (25.4)	186 (30.0)	N/A
Ex-drinker, n (%)	N/A	30 (1.6)	18 (2.4)	N/A	13 (0.8)	6 (0.9)	N/A	13 (1.0)	9 (1.8)	0 (0.0)	6 (1.0)	N/A
Never drinker, n (%)	N/A	1499 (81.5)	613 (83.1)	N/A	1486 (94.1)	656 (95.5)	N/A	1176 (91.7)	461 (91.7)	53 (74.6)	428 (69.0)	N/A
Regular exercise[c], n (%)	-	-	-	34.0 (20.1)	359.0 (22.7)	131.0 (19.1)	44 (37.0)	451 (35.2)	137 (27.2)	23 (32.9)	250 (40.2)	63 (29.3)
Food												
Total energy intake, kcal, mean (SD)	1746.2 (381.1)	1871.1 (422.3)	1880.2 (437.1)	1731.0 (358.1)	1808.5 (372.3)	1829.6 (412.5)	1697.3 (418.1)	1790.5 (500.5)	1786.8 (529.3)	1697.6 (424.2)	1807.6 (423.1)	1710.0 (433.4)
SFA, %E, mean (SD)	5.6 (1.4)	5.6 (1.5)	5.6 (1.5)	5.7 (1.5)	6.0 (1.5)	5.7 (1.4)	7.1 (2.6)	7.2 (2.7)	7.0 (2.5)	6.7 (2.6)	6.6 (2.4)	6.5 (2.5)
MUFA, %E, mean (SD)	7.4 (2.1)	7.4 (2.1)	7.6 (2.2)	7.7 (2.0)	8.0 (1.9)	7.7 (2.0)	7.9 (2.7)	8.0 (3.0)	7.9 (2.7)	8.5 (3.1)	8.3 (2.8)	8.4 (3.2)
PUFA, %E, mean (SD)	5.4 (1.6)	5.4 (1.5)	5.6 (1.6)	5.7 (1.6)	5.8 (1.5)	5.7 (1.5)	6.5 (2.2)	6.5 (2.1)	6.6 (2.1)	5.9 (2.0)	5.8 (2.0)	5.7 (2.2)

BMI, body mass index; MUFA, monounsaturated fatty acids; N/A, not avairable because of small sample size (≤5) included; PUFA, polyunsaturated fatty acids; SD, standard deviation; SFA, saturated fatty acids; TC, total cholesterol.

[a]BMI was categorized into underweight (<18.5 kg/m^2), normal (18.5–25.0 kg/m^2), overweight (≥25.0 kg/m^2).

[b]High TC was defined as ≥220 mg/dL.

[c]Regular exercise was defined as exercise ≥2 times/week and ≥30 min/session.

eTable 3. Characteristics of treated participants by BMI categories

Variables	1990			2000			2010		
	Underweight[a]	Normal[a]	Overweight[a]	Underweight[a]	Normal[a]	Overweight[a]	Underweight[a]	Normal[a]	Overweight[a]
Men									
n	≤5	48	23	≤5	88	54	≤5	72	66
Age, years, mean (SD)	N/A	65.0 (9.3)	61.6 (7.9)	N/A	66.0 (8.9)	65.1 (7.7)	N/A	69.3 (8.6)	68.3 (8.5)
TC, mg/dL, mean (SD)	N/A	211.5 (46.8)	229.8 (39.5)	N/A	210.7 (50.7)	206.0 (33.0)	N/A	191.2 (33.1)	187.1 (33.7)
High TC[b], n (%)	N/A	17 (35.4)	13 (56.5)	N/A	21 (0.4)	15 (0.4)	N/A	13 (18.1)	10 (15.2)
BMI, kg/m^2, mean (SD)	N/A	22.7 (1.8)	27.2 (1.4)	N/A	22.8 (1.5)	27.1 (2.0)	N/A	22.8 (1.8)	27.6 (2.6)
Smoking status									
Current and Ex-smoker, n (%)	N/A	34 (70.8)	N/A	N/A	18 (26.1)	12 (25.0)	N/A	54 (75.0)	58 (87.9)
Never smoker, n (%)	N/A	14 (29.2)	N/A	N/A	51 (73.9)	36 (75.0)	N/A	18 (25.0)	8 (12.1)
Drinking status									
Current drinker, n (%)	N/A	18 (37.5)	N/A	N/A	29 (0.4)	N/A	N/A	N/A	N/A
Ex-drinker, n (%)	N/A	10 (20.8)	N/A	N/A	14 (0.2)	N/A	N/A	N/A	N/A
Never drinker, n (%)	N/A	20 (41.7)	N/A	N/A	26 (0.4)	N/A	N/A	N/A	N/A
Regular exercise[c], n (%)	N/A	14 (29.2)	≤5	N/A	36 (0.5)	26 (0.5)	N/A	35 (48.6)	30 (45.5)
Food									
Total energy intake, kcal, mean (SD)	N/A	2104.3 (476.5)	2226.1 (348.9)	N/A	2116.4 (621.0)	2090.1 (683.4)	N/A	2092.8 (501.7)	2001.1 (451.6)
SFA, %E, mean (SD)	N/A	6.0 (1.3)	5.4 (1.5)	N/A	6.5 (2.5)	6.0 (2.1)	N/A	6.2 (2.4)	5.7 (2.0)
MUFA, %E, mean (SD)	N/A	7.9 (1.6)	7.5 (2.0)	N/A	7.5 (2.7)	7.1 (2.5)	N/A	7.8 (2.8)	7.4 (2.4)
PUFA, %E, mean (SD)	N/A	5.8 (1.5)	5.5 (1.4)	N/A	6.1 (1.9)	6.2 (2.0)	N/A	5.4 (1.7)	5.4 (1.9)
Women									
n	≤5	98	61	13	195	123	6	147	98
Age, years, mean (SD)	N/A	66.0 (8.7)	65.4 (7.6)	66.3 (11.6)	67.4 (8.7)	66.0 (7.9)	72.3 (8.5)	70.2 (8.1)	70.0 (7.4)
TC, mg/dL, mean (SD)	N/A	237.6 (40.7)	247.1 (44.0)	220.6 (23.9)	220.3 (34.1)	221.0 (32.0)	219.3 (26.5)	203.8 (27.0)	199.5 (31.1)
High TC[b], n (%)	N/A	59 (60.2)	42 (68.9)	≤5	79 (50.6)	51 (53.1)	≤5	36 (24.5)	22 (22.4)
BMI, kg/m^2, mean (SD)	N/A	22.6 (1.7)	27.6 (2.4)	17.6 (0.6)	22.4 (1.6)	27.6 (2.2)	17.7 (0.4)	22.1 (1.7)	27.5 (2.5)
Smoking status									
Current and Ex-smoker, n (%)	N/A	16 (16.3)	9 (14.8)	N/A	8 (4.5)	8 (6.8)	N/A	10 (6.8)	8 (8.2)
Never smoker, n (%)	N/A	82 (83.7)	52 (85.2)	N/A	169 (95.5)	109 (93.2)	N/A	137 (93.2)	90 (91.8)
Drinking status									
Current drinker, n (%)	N/A	N/A	N/A	N/A	N/A	N/A	0 (0.0)	N/A	N/A
Ex-drinker, n (%)	N/A	N/A	N/A	N/A	N/A	N/A	0 (0.0)	N/A	N/A
Never drinker, n (%)	N/A	N/A	N/A	N/A	N/A	N/A	6 (100.0)	N/A	N/A
Regular exercise[c], n (%)	N/A	23 (23.5)	20 (32.8)	≤5	58 (32.6)	40 (34.2)	≤5	62 (42.5)	29 (29.9)
Food									
Total energy intake, kcal, mean (SD)	N/A	1759.5 (346.2)	1771.0 (309.4)	1572.2 (405.2)	1735.7 (454.7)	1772.4 (531.5)	1917.5 (560.6)	1697.4 (428.5)	1704.0 (361.0)
SFA, %E, mean (SD)	N/A	6.0 (1.5)	5.7 (1.4)	7.5 (2.2)	7.0 (2.5)	6.7 (2.3)	7.0 (1.8)	6.4 (2.2)	6.1 (2.3)
MUFA, %E, mean (SD)	N/A	8.0 (2.0)	7.8 (1.9)	7.5 (3.1)	7.7 (2.6)	7.5 (2.5)	9.0 (1.2)	7.9 (2.7)	7.3 (2.5)
PUFA, %E, mean (SD)	N/A	5.9 (1.5)	5.8 (1.4)	5.6 (1.9)	6.5 (2.0)	6.4 (2.2)	6.8 (1.7)	5.4 (2.1)	5.5 (1.8)

BMI, body mass index; MUFA, monounsaturated fatty acids; N/A, not avairable because of small sample size (≤5) included; PUFA, polyunsaturated fatty acids; SD, standard deviation; SFA, saturated fatty acids; TC, total cholesterol.

[a]BMI was categorized into underweight (<18.5 kg/m^2), normal (18.5–25.0 kg/m^2), overweight (≥25.0 kg/m^2).

[b]High TC was defined as ≥220 mg/dL.

[c]Regular exercise was defined as exercise ≥2 times/week and ≥30 min/session.
